# Function of *desiccate* in gustatory sensilla of *drosophila melanogaster*

**DOI:** 10.1038/srep17195

**Published:** 2015-11-27

**Authors:** Takeshi Kawano, Masasuke Ryuda, Hitoshi Matsumoto, Masanori Ochiai, Yasunori Oda, Teiichi Tanimura, Gyorge Csikos, Megumi Moriya, Yoichi Hayakawa

**Affiliations:** 1 Department of Applied Biological Sciences, Saga University, Saga 840-8502, Japan; 2 The Analytical Research Center for Experimental Sciences of Saga University, Saga 840-8502, Japan; 3 Institute of Low Temperature Science, Hokkaido University, Sapporo 060-0819, Japan; 4 Department of Biology, Graduate School of Sciences, Kyushu University, Hakozaki, Fukuoka 812-8581, Japan; 5 Department of Anatomy, Cell and Molecular Biology, Eotvos Lorand University, Budapest, Hungary, H-1117.

## Abstract

*Desiccate* (*Desi*), initially discovered as a gene expressing in the epidermis of *Drosophila* larvae for protection from desiccation stress, was recently found to be robustly expressed in the adult labellum; however, the function, as well as precise expression sites, was unknown. Here, we found that *Desi* is expressed in two different types of non-neuronal cells of the labellum, the epidermis and thecogen accessory cells. Labellar *Desi* expression was significantly elevated under arid conditions, accompanied by an increase in water ingestion by adults. *Desi* overexpression also promoted water ingestion. In contrast, a knockdown of *Desi* expression reduced feeding as well as water ingestion due to a drastic decrease in the gustatory sensillar sensitivity for all tested tastants. These results indicate that *Desi* helps protect insects from desiccation damage by not only preventing dehydration through the integument but also accelerating water ingestion via elevated taste sensitivities of the sensilla.

Regulation of their water concentration is a fundamental requirement for all organisms. In particular, small terrestrial arthropods such as insects have an extremely large surface-to-volume ratio and are in danger of desiccation by evaporation through the integument to the environment. The conservation of body water is therefore essential for their survival^1^, and the wax layer coating the external surface of the integument certainly plays an indispensable role in water conservation^2^,^3^. Although holometabolous insect larvae have a much less lipidic cuticle, we do not know whether there is a desiccation-resistance system specific to the larval instar. Furthermore, various insect larvae show a drastic behavioral transition during the final instar: for example, *Drosophila* larvae remain immersed in the food source and feed constantly until the mid-third instar (foraging stage), when they enter a wandering stage, characterized by cessation of eating, purging of the gut, and exiting the food source to search for a suitable dry pupation site^4^,^5^. Because it is plausible that this behavioral change exposes larvae to desiccation stress, we speculated that larvae protect themselves by inducing a stage-specific desiccation tolerance. To clarify this question, we recently sought genes whose expression is enhanced in larvae by desiccation stress. We analyzed gene expressions in *Drosophila melanogaster* larvae in both foraging and wandering stages, and identified *CG14686,* whose expression was preferentially elevated in wandering stage larvae^6^. Furthermore, expression of this gene was also elevated in foraging larvae when they were placed in arid conditions. Overexpression of *CG14686* increased larval resistance to desiccation stress during the early foraging stage. *CG14686* RNAi larvae lost more weight under desiccated conditions than control larvae, and subsequently their mortality rates significantly increased. Based on these data, we dubbed this gene *Desiccate* (*Desi*). *Desi* encodes a 261-amino acid single-pass transmembrane protein with notable motifs, such as SH2 and PDZ domain-binding motifs and a cAMP-dependent protein kinase phosphorylation motif. Although the larval epidermis was initially identified as the primary tissue for *Desi* expression, our subsequent study of adults illustrated that gustatory sense organs of the labellum express *Desi* more robustly than the epidermis at this stage. Morphological analysis of *Desi* expression in the labellum roughly revealed that *Desi* was expressed in capsular layers surrounding the gustatory neurons^7^. Furthermore, we found that induction of forced cell death in *Desi*-expressing cells caused drastic mortality of the transgenic fly pharate adults with malformation of the labellum. Although these results imply the importance of *Desi-*expressing cells and Desi itself in the adult labellum, the functional role of labellar *Desi* as well as its precise expression sites remain unknown.

In the present study, we primarily sought to reveal the localization of *Desi* expression in the adult labellum and larval epidermis. Electron microscopic analyses of labellar *Desi* expression localized two different types of non-neuronal cells, epidermis and thecogen cells. Desi in the adult labellum epidermis showed similar localization as that in the larval body epidermis: Desi signals localized around the tips of microvilli on the apical surface of the epidermal cells and in the assembly zone between the epidermis and lamellate cuticle. Thecogen cells also produce Desi proteins and likely release them into the inner sensillum lymph sinus. The biological role of *Desi* expressing in the labellum was analyzed by manipulation as well as analyses of its expression levels. Labellar expression of *Desi* was elevated in *Drosophila* adults, which was accompanied by an increase in their water ingestion under arid conditions. This observation was consistent with the fact that *Desi* overexpression activated the water-seeking activity. In contrast, flies expressing RNAi against *Desi* significantly decreased their water ingestion due to desensitization of the labellar sensilla. These results indicate the essential role of *Desi* in regulating normal taste sensing by the gustatory organs, which is very important for animals to maintain an adequate water concentration by acceleration of water ingestion via elevation of the sensillar taste sensitivity, especially under arid conditions.

## Results

### Morphological analysis of *Desi* expression

**Figure 1:**
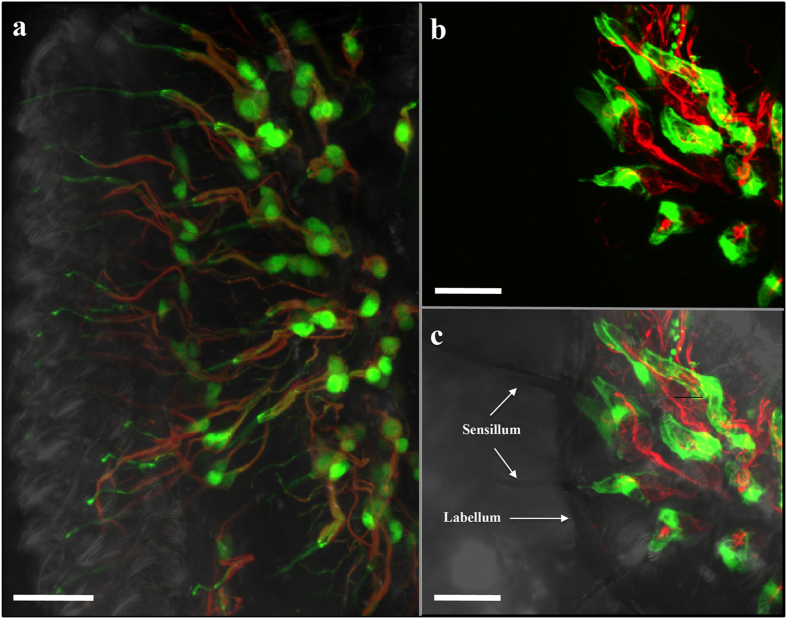
Morphological analyses of *Drosophila* labellar sensilla expressing *Desi.* (**a**) Composite image of transmitted-light and fluorescent images of labellar *Desi*-expressing cells (green fluorescence) and gustatory neuron dendrites (red fluorescence). GFP expression was driven by the *Desi-Gal4* driver. Scale bar indicates 20 μm. (**b**) Magnified fluorescent image of labellar *Desi*-expressing cells and gustatory neuron dendrites. Other explanations are as in (**a**). Scale bar indicates 40 μm. (**c**) Magnified composite image of transmitted-light and fluorescent images of labellar *Desi*-expressing cells and gustatory neuron dendrites. Other explanations are as in (**a**). Scale bar indicates 40 μm.

**Figure 2:**
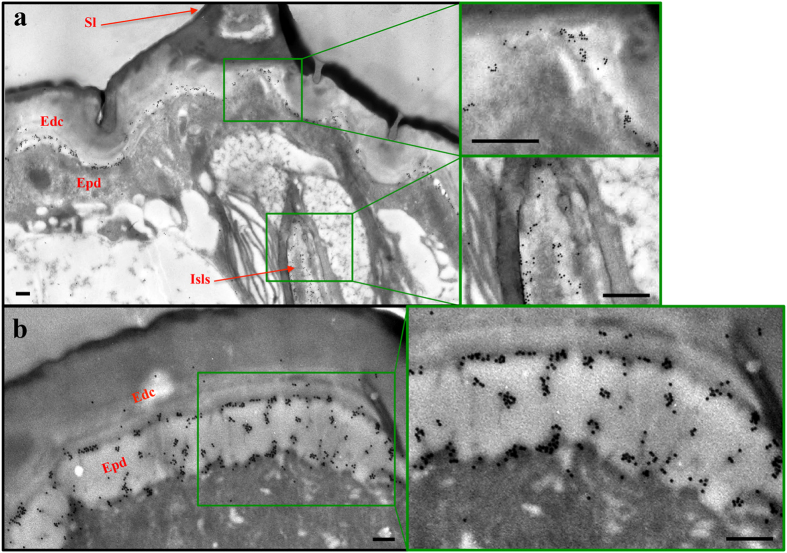
Electron microscopic analysis of Desi distribution in *Drosophila* labellum. (**a**) Broad scope of immunoelectron microscopic localization of Desi in the non-neuronal cells around the labellar sensillum of *Drosophila* flies. Thin sections of labella of control *UAS-dsDesi* flies were probed using anti-Desi IgG. Note that gold particles are distributed in two different regions, the assembly zone close to the epidermis (upper panel) and the inner sensillum lymph sinus (lower panel). Scale bars indicates 0.05 μm. Sl:Sensillum, Edc:Endocuticle, Epd:Epidermis, Isls:Inner sensillum lymph sinus. (**b**) Narrow scope of immunoelectron microscopic localization of Desi in the assembly zone between epidermis and endocuticle. Scale bars indicate 0.05 μm. Immunoelectron microscopic localizations of Desi in control fly sensillum using non-immunized IgG and in *Desi* RNAi fly using anti-Desi IgG were shown in Supplementary Fig. 2. Other explanations are as in (**a**).

**Figure 3:**
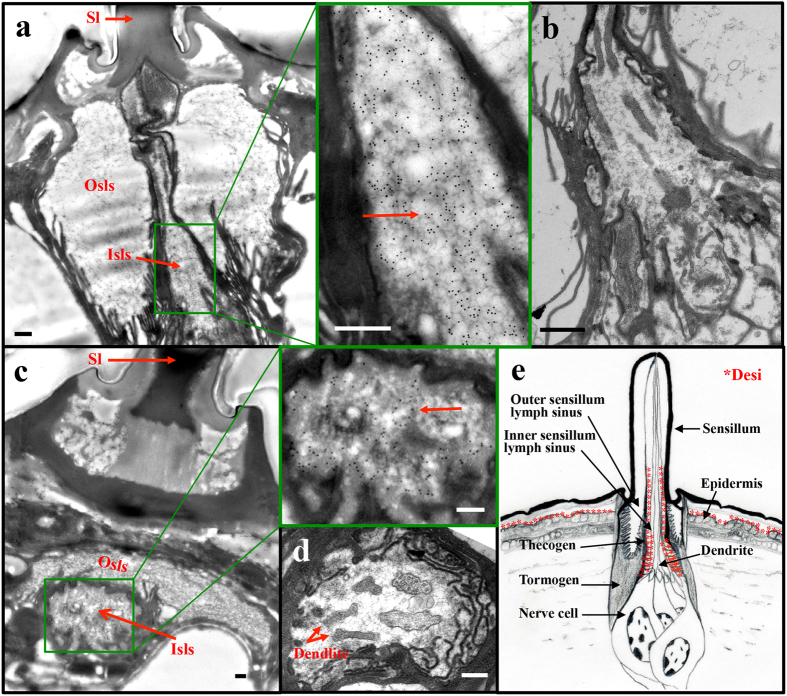
Electron microscopic analysis of Desi distribution in *Drosophila* labellum. (**a**) Broad and narrow scopes of immunoelectron microscopic localization of Desi in the longitudinal section of the inner sensillum lymph sinus. Thin sections of labella of control *UAS-dsDesi* flies were probed using anti-Desi IgG. Note that gold particles are distributed in the sinus. Scale bars indicate 0.05 μm. Many gold particles were observed in the region indicated with a red arrow. Sl:Sensillum, Edc:Endocuticle, Epd:Epidermis, Isls:Inner sensillum lymph sinus, Osls:Outer sensillum lymph sinus. (**b**) Conventional electron microscopic observation of the inner sensillum lymph sinus in the longitudinal section. Scale bar indicates 0.05 μm. (**c**) Broad and narrow scopes of immunoelectron microscopic localization of Desi in the transverse section of the inner sensillum lymph sinus. Note that the Desi signal distribution in this section together with that of the longitudinal section shows Desi distribution in the lymph. Scale bars indicate 0.1 μm. Other explanations are as in (**a**). (**d**) Conventional electron microscopic observation of inner sensillum lymph sinus in the transverse section. Scale bar indicates 0.1 μm. (**e**) Diagram of Desi distribution in two different regions of the labellum: the assembly zone between epidermis and endocuticle (partly on microvilli) and the inner sensillum lymph sinus (partly on thecogen cells). *shows Desi distribution.

In prior morphological analyses, we roughly observed labellar *Desi* expression in the region surrounding the gustatory neurons of *Drosophila melanogaster* adults. To identify the precise cell type expressing *Desi* in the labellum, we used a transgenic fly expressing GFP under the direction of the *Desi-Gal4* driver. Strong GFP signals were detected in the capsular layers covering the proximal dendrites and nerve cell bodies in the labellum (Fig. 1a). Higher magnification clearly distinguished Desi-expressing cells from gustatory neurons and dendrites (Fig. 1b,c). To confirm the distribution of Desi, immunoelectron microscopy was conducted using anti-Desi IgG. Gold particles were observed in a similar region, the inner sensillum lymph sinus, together with another region, the labellar epidermis (Fig. 2a, negative controls in Supplementary Fig. 1). Therefore, immunoreactive signals are distributed in two different regions: the labellar epidermis and inner sensillum lymph sinus. In the former region, gold particles were detected as small patches around the tips of microvilli on the apical surface of the epidermal cells and in the assembly zone^8^ between the epidermis and the lamellate endocuticle (Fig. 2b). A similar localization of Desi was observed in the larval body epidermis (Supplementary Fig. 2). In the latter region, gold particles were found to be localized to the thecogen cells of the inner lumen of the sensillum by both immunoelectron and conventional electron microscopic observations of longitudinal (Fig. 3a,b) and transversal sections (Fig. 3c,d) of the sensillum basement. Higher magnification of the observation in both the assembly zone (Supplementary Fig. 3) and the sensillum lymph sinus (Supplementary Fig. 4) showed that Desi-immunoreactive signals are not dispersed but form small clusters. Because Desi was initially characterized as a protein with a single-pass transmembrane domain, it is reasonable to interpret these morphological results showing that *Desi* is expressed in both labellar epidermis and thecogen cells as membrane proteins in the microvilli and thecogen cells, as summarized in the diagram (Fig. 3e).

### Effect of *Desi* RNAi on water ingestion

**Figure 4:**
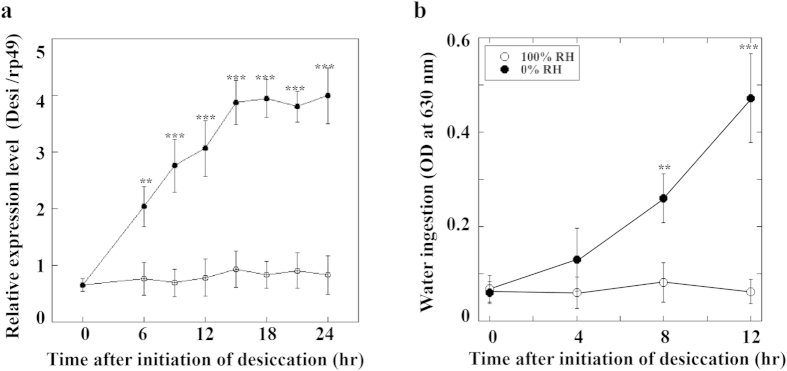
Labellar *Desi* expression levels and feeding activities of *Drosophila* adults under desiccation stress. (**a**) Real-time quantitative RT-PCR analysis of *Desi* expression in the labella with and without desiccation stress. *y w* strain adults were placed under 100% (open circle) or 0% (close circle) relative humidity (RH), and the labellar *Desi* expression levels were measured. Data are given as means ± SD for five separate measurements using 50 adults each. ** and *** denote *P* < 0.01 and 0.001 relative to values under 100% RH, respectively (Tukey’s HSD). (**b**) Water ingestion by *y w* strain adults was measured by putting test flies on 1.0% blue dye solution on absorbent cotton for 1 h after exposing them to wet (100% RH) or dry (0% RH) conditions for indicated periods^36^. Other explanations are as in (**a**).

We next sought to define the functional role of *Desi* expression in these regions of the labellum. Since we previously demonstrated that the elevation of epidermal *Desi* expression under arid conditions contributes to the protection of *Drosophila* larvae from desiccation stress^6^, we examined whether labellar *Desi* expression is also affected by humidity, like that in the larval epidermis. When *Drosophila y w* adults were placed under 0% relative humidity (RH), *Desi* expression levels in the labellum increased linearly for 15 h to approximately five times the initial level, indicating that *Desi* expression in the gustatory organs is also elevated by desiccation stress (Fig. 4a). We next performed a water ingestion assay on *y w* adults under arid (0% RH) and moist (100% RH) conditions. The amount of water consumed by the adults under 0% RH was much larger than that consumed under 100% RH, indicating that desiccation enhanced the water ingestion of the adults (Fig. 4b).

**Figure 5:**
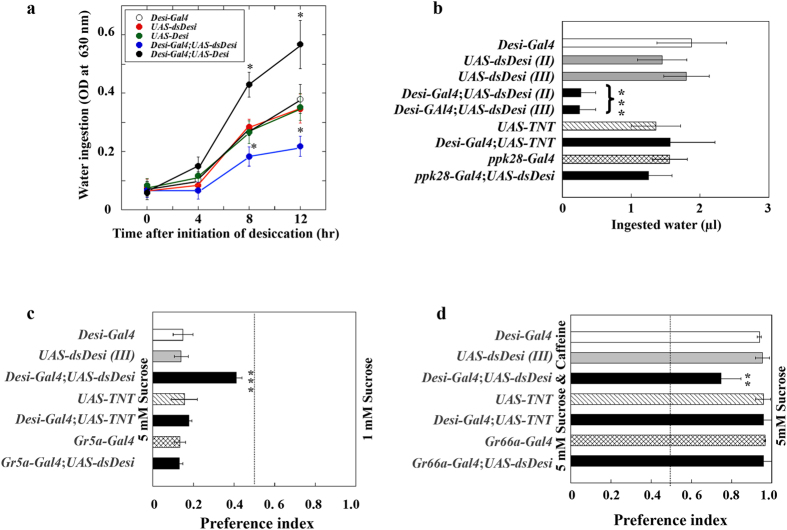
Water ingestion and feeding behaviors of control and *Desi* RNAi adults. (**a**) Water ingestion of transgenic fly lines was measured by putting test flies on 1.0% blue dye solution on absorbent cotton for 1 h after exposing them to 0% RH for indicated periods under 0% RH. Data are given as means ± SD for six separate measurements using 15 flies each. * and ** denote *P* < 0.05 and 0.01 relative to *Desi-Gal4* control (Tukey’s HSD). Two-day-old male adults were used. (**b**) Water ingestion by individual flies was measured by a CAFE assay. Data are given as means ± SD for five separate measurements using 20 adults each. *** denotes *P* < 0.001 relative to *Desi-Gal4* control (Tukey’s HSD). (**c**) Two-way choice assays were conducted using 1 mM and 5 mM sucrose. Male flies of control, *Desi*-RNAi (*Desi-Gal4*;*UAS-dsDesi,Gr5a-Gal4*;*UAS-dsDesi*), and *TNT*-overexpression lines (*Desi-Gal4*;*UAS-TNT*) were used for the assay. Other explanations are as in (**b**). (**d**) Two-way choice assays were conducted with 5 mM sucrose or 5 mM sucrose plus 5 mM caffeine. Male flies of control, *Desi*-RNAi (*Desi-Gal4*;*UAS-dsDesi,Gr66a-Gal4*;*UAS-dsDesi*), and *TNT*-overexpression lines were used for the assay.

To estimate the contribution of *Desi* to the water ingestion, we examined the effects of *Desi* overexpression and knockdown on the water ingestion of the adults. Because it was previously demonstrated that the *Desi* promoter*-Gal4* driver containing the 1,010-bp 5′ flanking region of *Desi* works preferentially in the gustatory organs^7^, this driver line was used for the tissue-specific manipulation of *Desi* expression (Supplementary Fig. 5). Overexpression of *Desi* under the direction of the *Desi-Gal4* driver elevated the water ingestion of test flies significantly after 8 h of desiccation stress (0% RH) (Fig. 5a). In contrast, a knockdown of *Desi* expression in the gustatory organs repressed water ingestion by test flies (Fig. 5a), indicating the possibility that expression levels of *Desi* in the labellum affect the water-sensing abilities of the labellar sensilla.

### Effect of *Desi* RNAi on tastant recognition

To examine the expression site as well as the functional role of *Desi*, we performed a different type of water ingestion assay, a CAFE assay, using several transgenic fly lines. When test flies were allowed to drink from a capillary tube, *Desi* RNAi flies consumed much less water than the control strain flies did (Fig. 5b). This reduction in water consumption was not observed in two other transgenic lines in which neurotoxin *tetanus toxin light chain (TNT)* and *dsDesi* are respectively expressed in *Desi-*expressing cells and sensory neurons, *Desi-Gal4*;*UAS-TNT* and *pickpocket28* (*ppk28*)*-Gal4*;*UAS-dsDesi,* suggesting that neither neurotoxin overexpression in *Desi-*expressing cells nor *Desi* RNAi in sensory neurons caused any change in the water ingestion (Fig. 5b). These data confirmed our previous preliminary observation that *Desi* is expressed in non-neuronal cells^7^. No visible difference between control and *Desi* RNAi flies was found in labellar neurons as well as labellar sensilla themselves, at least by simple observation (Supplementary Figs 6 and 7). Furthermore, RT-PCR demonstrated normal expression levels of several neuron-specific genes, including gustatory receptor genes, in the labellum of *Desi* RNAi flies (Supplementary Fig. 7). These results suggested that the sensitivities of labellar neurons were severely disturbed by a knockdown of *Desi* expression in such non-neuronal cells, although *Desi* RNAi did not affect the morphological properties of labellar neurons because *Desi* is expressed in non-neuronal labellar cells. Thus, a change in Desi expression must affect indirectly the neuronal functions of taste sensilla.

Prior studies located *Desi* expression sites in most adult gustatory organs including the labellum, wings, and tarsi^7^. This observation allowed us to expect the plausible contribution of Desi to sense various tastants besides water. To evaluate whether the sensory disorders in *Desi* RNAi flies are limited to water detection, we analyzed their abilities to detect other tastants such as sugar and caffeine using a two-way choice test. When presented with a choice between 1 mM and 5 mM sucrose, control flies displayed a very strong preference for 5 mM sucrose, but in contrast, *Desi* RNAi flies did not (Fig. 5c). Furthermore, the choice test was used to check the avoidance of caffeine. The result showed a significant reduction in the avoidance by *Desi* RNAi flies (Fig. 5d). The preference for sucrose and distaste for caffeine were not changed in the transgenic lines in which TNT was overexpressed in *Desi-*expressing cells (*Desi-Gal4*;*UAS-TNT*) or *Desi* knockdown under the direction of taste receptor gene drivers such as sucrose (*Gr5a-Gal4*;*UAS-dsDesi*) or caffeine (*Gr66a-Gal4*;*UAS-dsDesi*) receptor gene drivers, indicating that neurotoxin overexpression in *Desi-*expressing cells did not affect taste sensing and that *Desi* expression in *Desi-*expressing cells themselves is important in preserving normal taste recognition in test flies. The contribution of Desi to the detection of various tastants was further confirmed by showing that *Desi* overexpression and knockdown increased and decreased, respectively, feeding activities of test transgenic flies especially under arid conditions (Supplementary Fig. 8). Furthermore, the *Desi* RNAi-induced decline in feeding activities was also observed in test adults even under 100% RH (Supplementary Fig. 8), and consequent extension of the life span was detected (Supplementary Fig. 9).

### Effect of *Desi* RNAi on tastant-response behavior

**Figure 6:**
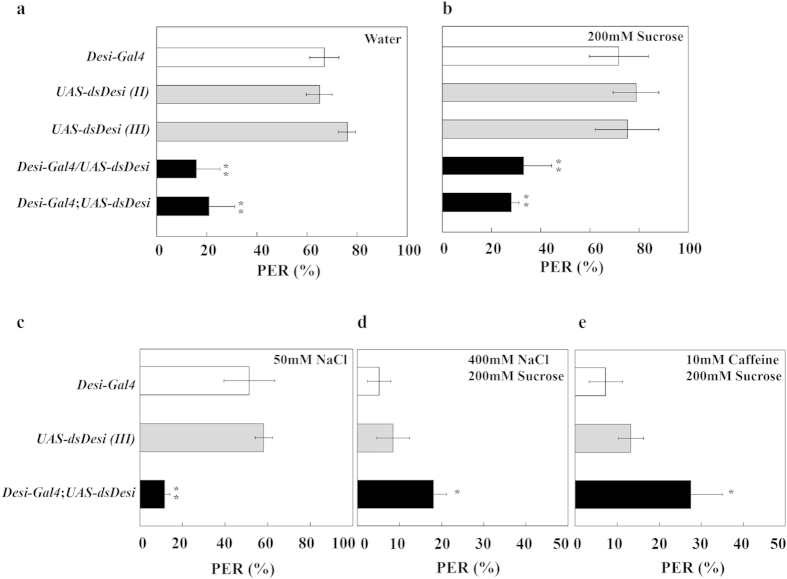
Tastant responses of control and *Desi* RNAi adults. (**a**) Response to water of control and *Desi* RNAi adults. Data are given as means ± SD for five separate measurements using 50 adults each. ** denotes *P* < 0.01 relative to *Desi-Gal4* control (Tukey’s HSD). (**b**) Response to sucrose of control and *Desi* RNAi adults. Other explanations are as in (**a**). (**c**) Response to the low concentration of NaCl of control and *Desi* RNAi adults. Other explanations are as in (**a**). (**d**) Aversion of control and *Desi* RNAi adults to the high concentration of NaCl. Other explanations are as in (**a**). (**e**) Aversion of control and *Desi* RNAi adults to caffeine. Only male flies were used for tests. * denotes *P* < 0.05 relative to *Desi-Gal4* control (Tukey’s HSD). Other explanations are as in (**a**).

To verify the contribution of Desi to gustatory sensing, we examined the ability of transgenic flies to produce a tastant response following stimulation of tarsi with various tastants by the proboscis extension reflex (PER) test. When one prothoracic leg of water-deprived test transgenic flies was touched with water, the proportion of *Desi* RNAi flies who responded to water was found to be much lower than that of control flies (Fig. 6a). Depression of the tastant-induced response of *Desi* RNAi flies was also observed when water was replaced by 200 mM sucrose or 50 mM NaCl (Fig. 6b,c). Furthermore, when the leg was exposed to 200 mM sucrose solution containing aversive compounds such as 400 mM NaCl (Fig. 6d) or 10 mM caffeine, avoidance of these compounds was distinctly visible in control flies but was significantly impaired in *Desi* RNAi flies (Fig. 6e). The tastant-induced PER reaction did not change in the transgenic lines in which *TNT* was overexpressed in *Desi*-expressing cells (*Desi*-*Gal4*;*UAS-TNT*) (Supplementary Fig. 10) or *Desi* knockdown under the direction of gustatory receptor gene drivers such as *ppk28-Gal4* (water), *Gr5a-Gal4* (trehalose), and *Gr66a* (caffeine) (Supplementary Fig. 11). These results demonstrated that neither neurotoxin expression in *Desi*-expressing cells nor *Desi* RNAi in neuronal cells affected gustatory sensing of test flies. Based on these data, it is reasonable to conclude that the wild-type function of Desi in non-neuronal *Desi*-expressing cells is required for robust detection of tastants, including water.

### Effect of *Desi* RNAi on electrophysiological responses to tastants

**Figure 7:**
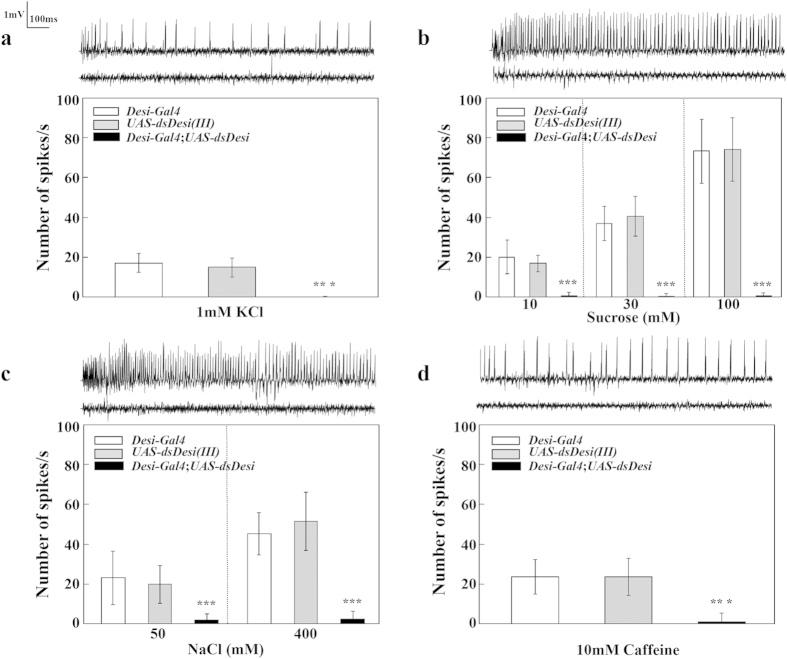
Neuronal response of control and *Desi* RNAi adults to various tastants. (**a**) Sample tip recordings of sensilla and average frequencies of action potentials of control and Desi RNAi (*Desi-Gal4*;*UAS-dsDesi*) adults in response to water (1 mM KCl). Data are given as means ± SD for 20–31 separate measurements using 20 adults. *** denotes *P* < 0.001 relative to *Desi-Gal4* control (Tukey’s HSD). Upper and lower traces above the graph show the actual recording of control (*UAS-dsDesi)* and *Desi* RNAi flies, respectively. (**b**) Sample tip recordings of sensilla (stimulated by 100 mM sucrose) and average frequencies of action potentials of control and *Desi* RNAi adults in response to sucrose. Data are given as means ± SD for 20–30 separate measurements using 20 adults. Other explanations are as in (**a**). (**c**) Sample tip recordings of sensilla (stimulated by 400 mM NaCl) and average frequencies of action potentials of control and *Desi* RNAi adults in response to NaCl. Data are given as means ± SD for 21–40 separate measurements using 21 adults. Other explanations are as in (**a**). (**d**) Sample tip recordings of sensilla and average frequencies of action potentials of control and *Desi* RNAi adults in response to 10 mM caffeine. Data are given as means ± SD for 20–36 separate measurements using 20 adults. Other explanations are as in (**a**).

To determine whether the electrophysiological response to tastants was changed by *Desi* RNAi, we initially measured water (1 mM KCl)-induced action potentials in the gustatory receptor neurons of the labellum sensillum by performing tip recordings. Consistent with the behavioral assays, the frequencies of action potentials were increased in control flies by application of water (Fig. 7a). In contrast, water-induced action potentials were virtually eliminated in *Desi* RNAi flies. Sucrose- and NaCl-induced action potentials were also observed in control flies and increased with increasing concentrations of both tastants, while *Desi* RNAi significantly eliminated their action potentials (Fig. 7b,c). Furthermore, caffeine-induced action potentials were detected in control flies but not in *Desi* RNAi flies (Fig. 7d). These data strongly indicate that *Desi* RNAi flies had lost their ability to detect all tastants tested in this study.

### Effect of *Desi* RNAi on water concentration in sensillum

**Figure 8:**
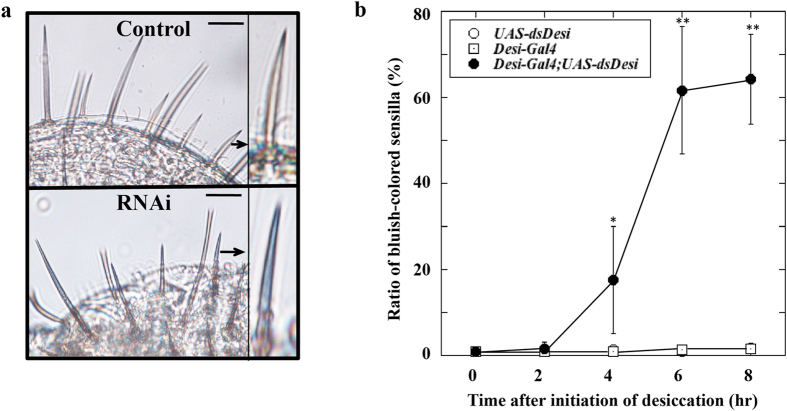
Analyses of water concentration in adult labellar sensilla. (**a**) CoCl_2_ incubation induced a bluish coloration in the labellar sensilla of *Desi* RNAi but not in those of control *UAS-Desi* adults. Whole bodies of flies that had been exposed to 0% RH for 6 h, were soaked in 1M CoCl_2_ ethanol solution for 1 h. Note that incubation of test flies in 1 M CoCl_2_ ethanol solution colored the sensilla of *Desi* RNAi flies but not the sensilla of control flies. Another control, *Desi-Gal4* flies, showed the same results as those of *UAS-Desi*. Each bar indicates 10 μm. Arrows indicate magnified sensilla on each labellum. (**b**) Ratio of bluish-colored sensilla on the labellum of control *UAS-Desi* and *Desi* RNAi flies that had been exposed to 0% RH for indicated periods. Ratios of bluish-colored sensilla on the labellum of *Desi* RNAi flies were less than 3% as long as they had been under 100% RH. * and ** denote *P* < 0.05 and 0.01 relative to *UAS-Desi* (Tukey’s HSD). Data are given as means ± SD for 24 separate measurements using 2 adults each. Other explanations are as in (**a**) except for the period of exposure to desiccation.

The *Desi* RNAi-induced defect in the taste sensory ability of the labellar sensilla led us to speculate that changes in the physical properties of labellar sensilla by which the water concentration in the inner sensillar sinus surrounding the dendrites of the gustatory neurons could be changed. To assess this possibility, we measured the water concentration in the inner sensillar sinus using CoCl_2_ ethanol solution because it has been reported that CoCl_2_ is useful for determination of water concentration^9^. We exposed control and *Desi* RNAi flies to 0% RH for 6 h, then dipped their whole bodies in 1 M CoCl_2_ ethanol solution for 1 h and compared the colors of the labellar sensilla (Fig. 8a). While there was no change in the color of the sensilla of control flies, the sensilla of *Desi* RNAi flies turned blue. The ratio of bluish sensilla on the labellum of *Desi* RNAi flies was significantly increased with the time of exposure of the flies to desiccation stress (Fig. 8b). Significant bluish coloration of the labellar sensilla was not observed in *Desi* RNAi flies when they had been placed under 100% RH, suggesting that *Desi* RNAi exposed to desiccation stress decreased the water concentration inside the sensilla because CoCl_2_ hydrated with water does not turn blue but red. Therefore, these observations can be interpreted to mean that Desi contributes to regulation of the aqueous environment inside the labellar sensillum.

## Discussion

In the present study, we characterized *Desi* expression in the labellum of *Drosophila* adults to define its functional roles as well as identify expressional sites. *Desi* expression levels in the labellum were significantly elevated by desiccation stress, accompanied by an increase in water ingestion by test flies, suggesting the possibility that *Desi* expression levels affect water ingestion. Phenotypic analysis using transgenic flies with manipulated *Desi* expression supported this interpretation. Overexpression of *Desi* under the direction of the *Desi-Gal4* driver elevated water ingestion of test flies compared with control flies. In contrast, *Desi* RNAi flies ingested significantly less water, and the loss of such behavioral responses was enhanced in test transgenic flies that had been exposed to desiccation stress before receiving water. These data indicate that *Desi* expression levels, which are crucially affected by humidity, are linearly correlated with the water ingestion of adult flies. Behavioral and electrophysiological analyses showed that the gustatory sensilla of *Desi* RNAi flies had significantly impaired ability to sense all tested tastants, water, sugar, salt, and caffeine. These results indicate the biological significance of *Desi* gene products for sensing tastants. A labellar sensillum is generally composed of two to four gustatory receptor neurons and one mechanoreceptor neuron whose dendrites are bathed in the sensillum lymph stored in the inner sinus (lumen) encapsulated by thecogen accessory cells^10^,^11^,^12^,^13^,^14^; therefore, the composition and volume of the sensillum lymph must be important for normal neuronal activity^15^. The labellar epidermal cells expressing Desi externally surround the inner sensillum lymph sinus that is also encapsulated by Desi-expressing thecogen cells. This means that the inner sensillum lymph is covered by two different Desi-expressing cell layers. If these Desi-expressing cells do not function properly, the lymph component could be altered, especially under arid conditions. This possibility was at least partly supported by the physiological observations: the labellar sensilla acquired a more bluish color in *Desi* RNAi flies compared with control flies when test flies were soaked in CoCl_2_ ethanol solution after exposure to desiccation (Fig. 5b). Therefore, it is reasonable to assume that *Desi* expressing in the labellum of the fly is essential to maintain the normal internal environment for the gustatory neurons in the labellum.

Studies of the regulation of *Drosophila* gustatory behaviors in the last decade have identified various types of genes contributing to the regulation of their feeding activities: gustatory receptor genes^16^,^17^,^18^,^19^,^20^,^21^,^22^,^23^, neuropeptide genes including insulin/insulin-like peptide genes^24^,^25^,^26^, and signal transduction component genes^27^,^28^. Although expression levels of these genes affect feeding activities of *Drosophila* flies, *Desi* falls into none of these gene categories. It was recently reported that odorant-binding proteins (OBPs) affect taste perception and host plant preference: while *Drosophila sechellia* shows a preference for the ripe fruit of *Morinda cirifolia*, its closely related species, *D. melanogaster*, avoids the fruit. However, introduction of the odorant-binding protein genes, *D. sechellia Obp57d* and *Obp57e*, shifted the oviposition-site preference of *D. melanogaster Obp57d/e*^*KO*^flies to that of the original species^29^. These results indicate the significance of the presence of the sensillum lymph as well as the OBPs because these proteins occur in the lymph of olfactory sensilla. Although the sensillum lymph in the gustatory sensilla has not been analyzed well as that in olfactory sensilla has, the present study implies its important responsibility for gustatory sensing.

The present morphological analyses showed the unique localization of Desi molecules in two different regions: the assembly zone (between the epidermis and endocuticle) and the inner sensillum lymph sinus. The fact that *Desi* encodes a 261-amino acid single-pass transmembrane protein points us to the future study concerning its functional properties as a membarane protein. Although we do not have any direct evidence that Desi regulates the water permeability of the epidermis and thecogen cell layer, the fact that immunoreactive Desi signals form small clusters around the tips of microvilli on the apical surface of the epidermal cells (Fig. 2b, Supplementary Fig. 3) and in the sensillum lymph sinus (Supplementary Fig. 8) enabled us to hypothesize a “specialized structure” composed of several Desi molecules or Desi molecules with other proteins that function as a transporter or channel. Prior study showed that the Desi molecule contains several conserved motifs, such SH2 and PDZ domain-binding motifs and a cAMP-dependent protein kinase phosphorylation site^6^. Among these motifs, the PDZ domain-binding motif has been found in various types of transporter and channel genes^30^. PDZ domain-containing proteins have been reported to play several critical roles, especially in (i) subcellular localization of transporters, (ii) stabilization of expression of transporters in the apical or basolateral membrane, and (iii) functional regulation via protein-protein interaction with transporters^31^,^32^. For example, it has been reported that the sarcolemmal localization of a water channel protein, aquaporin-4, *in vivo* depends on the presence of a dystrophin-bound α-syntrophin PDZ domain^33^. Based on these reports, it may be reasonable to assume that Desi functions as a protein organizing a transporter or channel in the non-neuronal cells around the labellar sensilla. This story may be also true for the epidermal cells in the larval integument because Desi expressing in the larval epidermis and the adult labellum shares a number of similarities in terms of the physiological importance and distribution^6^. The fact that the PDZ binding motifs are completely conserved in orthologous genes identified in a broad range of insect species, such as mosquitoes, bees, and beetles, also encourage us to do further investigation to test this prediction^6^.

Finally, based on the present results together with the conservation of orthologous genes in a broad range of insect species, we propose that the gene *Desi* contributes to protection of insects from desiccation risks due to preservation of body water in two different ways, protection of dehydration through the body integument and acceleration of water ingestion via elevation of the sensillar taste sensitivity.

## Methods

### Flies

*UAS-Desi* and *Desi-Gal4* lines were generated as described previously^6^,^7^. The RNAi stock (*UAS-dsDesi* (*dsCG14684*)) strains were obtained from the Vienna Drosophila RNAi Center (Vienna, Austria). The *Gr5a-Gal4* strain was provided by J. R. Carlson (Yale University, USA)^34^. *ppk28-Gal4* was provided by K. Scott (University of California-Berkeley, USA)^35^. *Gr66a-Gal4* is available from the Bloomington Center. *UAS-TNT* strains were gifts from C. O’Kane (University of Cambridge, Cambridge, UK). Mostly male flies were used experiments except as specifically described, and all analytical experiments were done at 25°C. All flies were kept in the culture tubes (inside humidities: over 80% RH) before using them as test animals. Arid condition (0% RH) was made in a Petri dish inside a desiccator containing dried silica gels, while moist condition (100% RH) was made in a Petri dish containing water-absorbed cotton.

### Quantification of feeding activity

The amount of medium ingested by test flies was quantified by measuring the absorbance of the whole body extract at 630 nm after feeding them the test diet containing 1.0% blue dye as described previously^36^.

### Analysis of water concentration in *Drosophila* labellum

The water concentration was measured basically according to the procedure of Ayres and Glanville^37^. Control (*Desi-Gal4* and *UAS-dsDesi*) and *Desi* RNAi flies were placed under 0% RH for indicated periods, and whole bodies were soaked in 1 M CoCl_2_ ethanol solution for 1 h. Labella of test flies were dissected, immediately rinsed in ethanol, and put on glass slides in glycerol. Sensilla of each labellum were observed using a confocal microscope (EZ-Ti system, Nikon). The percentage of bluish sensilla was scored by counting 10 sensilla from a randomly selected portion on each labellum. Sensilla were scored as bluish if they transmitted less than 30% of the visible light measured at 655 nm using the confocal software EZ-C1.

### Behavioral assays of feeding activity

Feeding behaviors were assayed by three different methods, a two-way choice assay^38^,^39^, capillary feeding (CAFE) assay^40^, and proboscis extension reflex (PER) test^41^ as described in Methods of the Supplementary Information.

### Electrophysiology

Electrophysiology was performed according to a procedure slightly modified from the tip-recording method^42^,^43^. Flies 1–2 days old were transferred to fresh medium 1 d before the experiment. To record activity from labellar taste neurons, flies were electrically grounded by inserting a glass capillary tube filled with *Drosophila* Ringer’s solution into the abdomen, and a recording electrode (glass capillary with a 20-μm diameter tip) filled with a test taste solution covered the tip of a single taste sensillum. Test sensilla were stimulated up to 2 s with the recording electrode, and electrical signals were amplified and recorded after filtration. The electrophysiological responses to sugar, salt, and water were recorded on an l-type sensillum, and those to caffeine were recorded on an i-type sensillum. Data were then analyzed with DBWAVE software^44^. Spikes were detected and analyzed by using interactive procedures in a custom software package, DBWAVE. Solutions of 1 mM KCl, 10–100 mM sucrose, 50–400  mM NaCl, and 10 mM caffeine were used to stimulate the water-, sugar-, salt-, and bitter-response neurons, respectively.

### Morphological analyses

Fluorescences of GFP and Alexa Fluor dye were monitored by a laser scanning confocal microscope (EZ-Ti system, Nikon). Sensory neurons in test flies were visualized by immunohistochemistry using the monoclonal antibody anti-Futsch:22C10, which is specific for the neuronal microtubule protein. Detailed procedures of electron microscopy and scanning electron microscopy are described in Methods of the Supplementary Information.

## Additional Information

**How to cite this article**: Kawano, T. *et al.* Function of *desiccate* in gustatory sensilla of *drosophila* *melanogaster*. *Sci. Rep.* **5**, 17195; doi: 10.1038/srep17195 (2015).

## Supplementary Material

Supplementary Information
