# Supplementary material for: Function of desiccate in gustatory sensilla of drosophila melanogaster
Source: Sci Rep. 2015 Nov 27;5:17195. doi: 10.1038/srep17195 (PMC4661605; doi:10.1038/srep17195)

Function of *Desiccate* in gustatory sensilla of *Drosophila melanogaster*

Takeshi Kawano[1], Masasuke Ryuda[2], Hitoshi Matsumoto[1], Masanori Ochiai[3], Yasunori Oda[1], Teiichi Tanimura[4], Gyorge Csikos[5], Megumi Moriya[3], Yoichi Hayakawa[1*]

[1]Department of Applied Biological Sciences, Saga University, Saga 840-8502, Japan; [2]The Analytical Research Center for Experimental Sciences of Saga University, Saga 840-8502, Japan; [3]Institute of Low Temperature Science, Hokkaido University, Sapporo 060-0819, Japan; [4]Department of Biology, Graduate School of Sciences, Kyushu University, Hakozaki, Fukuoka 812-8581, Japan; [5]Department of Anatomy, Cell and Molecular Biology, Eotvos Lorand University, Budapest, Hungary, H-1117.

*To whom correspondence should be addressed.
Tel/Fax: 81 952 28 8747; E-mail: hayakayo@cc.saga-u.ac.jp

Supplementary Information

Methods

Animals - *Drosophila* larvae and adults were reared on artificial food containing 8.7%(w/w) cornmeal, 5.2%(w/w) glucose, 3.5% (w/w) dried yeast, 0.3% antiseptic reagents, and 0.8%(w/w) agar, at $25\pm1^{\circ}C$[1]. The transgenic strains *UAS-Desi* and *Desi-Gal4* were generated as described previously[2].

Two-way Choice Assay - A two-choice preference test was performed by using modifications of a previously described procedure[3, 4]. For each assay, 50 flies (3 days old) were starved for 24 h on 1% agarose and then introduced into a 96-well microtiter dish filled with two types of test mixtures in alternate wells. Each test mixture contained 1% agarose, either blue or red dye, and an indicated tastant such as sucrose (for attraction test) and sucrose plus caffeine (for avoidance test). After allowing the flies to ingest freely for 60 min at room temperature in the dark, the animals were immediately frozen in the plates at -20°C, and the numbers of flies with blue (N^B), red (N^R), or purple (N^P) abdomens were assessed by visual inspection. The preference index (PI) values were calculated according to the following equation: PI = $(N^B + 0.5N^P)/N^{Total}$ or $(N^R + 0.5N^P)/N^{Total}$. PI values close to 1.0 or 0 indicate a significant preference for one or the other tastant, and the values close to 0.5 show a lack of preference. The dyes did

not cause preference changes, because no difference in PIs was caused by switching the dyes. Every measurement was repeated four times.

Capillary Feeding Assay - The capillary feeding (CAFÉ) assay was performed according to the slightly modified procedure of William *et al.*[5]. To test the water responses, flies were placed in a dry chamber over silica gel for 10 h. The assay chamber, containing the flies, was prepared using a 5 cm diameter glass vial to 15 cm length. Calibrated glass micropipettes (5 μl, VWR, West Chester, PA) filled with water by capillary action were inserted through the assay chamber's cap via truncated 200 μl pipette tips, and a mineral oil overlay (0.1 μl) was used to minimize evaporation. Every assay was conducted at 25°C for 30 min and repeated five times.

Proboscis Extension Reflex Test - The proboscis extension reflex (PER) test was carried out according to the slightly modified procedure of Kimura *et al.* [6]. We starved flies at 25°C for 24 h before allowing them free access to water (or indicated tastant solution), and then fixed each fly with myristyl alcohol on a plastic plate. To test the PER responses to test tastants, we incubated the fixed flies for 1 h in a humid chamber before stimulation with tastants. Every measurement was repeated five times.

RT-PCR analysis - Total RNA was extracted from whole bodies or indicated tissues using TRIzol (Gibco-BRL) according to the manufacturer's protocol. To determine whether the *Drosophila melanogaster* genes are expressed in each species of larvae and adults, RT-PCR was conducted essentially according to the procedure described previously[7]. First-strand cDNA was synthesized with oligo $(dT)_{12\text{-}18}$ primer using ReverTra Ace RT-PCR kit (Toyobo) according to the manufacturer's protocol. PCR amplification was performed with the following specific primer pairs: *Desi*-specific primer pair, GATAGCCATAAGTTCTATGCG and GCCTCCTTAATAGCCGTTCC; *rp49*-specific primer pair, GATCGTGAAGAAGCGCACCAAG and CCGGATTCAAGAAGTTCCTGGTG; *Elav*-specific primer pair, TAAAGGCGCCAACCTTTATG and AGGCAATGATAGCCCTTGTG; *Tau*-specific primer pair, GCACTGACAACACCACCAAC and TCGTTTTGCATTGTGTGGT; *Gr5a*-specific primer pair, AACTCTGGCCTGGATTGATG and CTTCTTCGTGGGCAGAAGTC; *Gr66A*-specific primer pair, ATGGTCTCCGACCAGTTGAC and AGGTGCTCACCAAAATGGAC; *ppk28*-specific primer pair, CCTCCATACTGACGGACGAT and GTGCGCGGAAAGTAGAACTC;

ppk11-specific primer pair, GGTCCGTGTCCGTGTCTACT and CGTGATTCCAACCCTCTGAT. PCR analyses of *Desi* gene expression levels in the transgenic flies were performed with the above primers using a modified profile as follows: 25 cycles of 30 sec at 95°C, 1 min at 55°C and 1 min at 72°C.

Real-time quantitative PCR analysis - Real-time quantitative PCR analysis of *Desi* expression in *D. melanogaster* larvae was carried out by using the Light-Cycler 1.3 instrument and software (Roche Applied Science) as described previously[8]. PCR specificity was confirmed by the molecular masses of the PCR products and melting curve analysis at each data point. The copy numbers of RNA coding the genes of interest were standardized against that of the RNA coding *rp49* in each sample.

Immunoelectron microscopy - *Drosophila* adult labella and larval integuments were isolated and fixed for 18 to 20 h in a mixture of 4 % paraformaldehyde, 0.2 % glutaraldehyde and 0.1 M sucrose, buffered by 0.05 M PIPES (pH 7.2). Each specimen was then washed in 0.05 M PIPES buffer containing 0.1 M sucrose, dehydrated through an ethanol series, and embedded in LR-Gold (London Resin Company Ltd., Berkshire, England). Polymerization was performed within a refrigerator at -5°C under UV light inside closed gelatin capsules. Fixed tissues were thin-sectioned with glass knives and placed on Formvar-coated nickel grids (100-mesh). Specimens were rinsed with Tris buffered saline (10 mM Tris-HCl, 0.9% NaCl, pH 7.4; TBS) containing 50% fetal bovine serum (FBS) (Dainippon Pharmaceutical, Osaka) and incubated for 1 h at 25°C with anti-Desi IgG (15 μg/ml of 50% FBS-TBS). After a thorough washing in TBS containing 0.1% Tween 20 (T-TBS), the specimens were incubated for 12 h at 25°C in goat-rabbit IgG conjugated to colloidal gold particles (10 or 15 nm; British Biocell International) with T-TBS. The grids were washed with T-TBS and distilled water and dried. Finally, they were stained with 2% uranyl acetate. For control, thin sections were treated with non-immunized rabbit IgG.

For conventional electron microscopy, labella dissected from test flies were immersed in cold fixative. Primary fixation was performed for 2 h at 4°C in 2% glutaraldehyde in 0.1 M cacodylate buffer containing 0.02M sucrose and 2 mM $CaCl_2$ (pH 7.4). The tissue was washed in sucrose/cacodylate buffer then post-fixed for 2 h in 1% OsO4 in veronl acetate buffer (pH 7.4) at 4°C, dehydrated in ethanol, immersed in epoxy propane and embedded in Araldite. Sections were stained with uranyl acetate and lead citrate and examined in a JEOL 100C electronmicroscope.

Scanning electron microscopy - *Drosophila* adults were fixed in 5.0 % glutaraldehyde in phosphate-buffered saline (PBS) (8 mM Na2HPO4, 1.5 mM KH2PO4, 137 mM NaCl, 2.7 mM KCl, pH 7.2). Following dehydration in ethanol and critical point drying, samples were gold coated and examined using a HITACHI S-3400N scanning electron microscope.

References

1. Ryuda, M., Tsuzuki, S., Tanimura, T., Tojo, S., Hayakawa, Y. A gene involved in the food preferences of larval *Drosophila melanogaster. J. Insect Physiol.* **54**, 1440-1445 (2008).
2. Kawano, T., Matsumoto, H., Maekawa, E., Nakano, F., Kanuka, H., Tsuzuki, S.*, et al.* Cells expressing Desiccate are essential for morphogenesis of labial sensilla in Drosophila melanogaster adults. *Entomol. Sci.* **14**, 183-191 (2011).
3. Moon, S.J., Kottgen, M., Jiao, Y., Xu, H., Montell, C. A taste receptor required for the caffeine response *in vitro. Curr. Biol.* **16**, 1812-1817 (2006).
4. Tanimura, T., Isono, K., Takamura, T., Shimada, I. Genetic dimorphism in the taste sensitivity to trehalose in *Drosophila melanogaster. J. Com.p Physiol.* **A147**, 433-437 (1982).
5. Ja, W.W., Carvalho, G.B., Mak, E.M., de la Rosa, N.N., Fang, A.Y., Liong, J.C., Brummel, T., Benzer, S. Prandiology of Drosophila and the CAFE assay. *Proc. Natl. Acad. Sci. USA* **104**, 8253-8256 (2007).
6. Kimura, K., Shimozawa, T., Tanimura, T. Isolation of Drosophila mutants with abnormal proboscis extension reflex. *J. Exp. Zool.* **239**, 393-399 (1986).
7. Ninomiya, Y., Kurakake, M., Oda, Y., Tsuzuki, S., Hayakawa, Y. Insect cytokine growth-blocking peptide signaling cascades regulate two separate groups of target genes. *FEBS J.* **275**, 894-902 (2008).
8. Kawano, T., Shimoda, M., Matsumoto, H., Ryuda, M., Tsuzuki, S., Hayakawa, Y**.** Identification of a gene, Desiccate, contributing to desiccation resistance in Drosophila melanogaster. *J. Biol. Chem.* **285**, 38889-38897 (2010).

Supplementary Figure Legends

Fig. 1. Immunoelectron microscopic analysis of Desi distribution in the labellum. A, Immunoelectron microscopic localization of Desi in the labellar sensillum of *Drosophila y w* fly using non-immunized IgG. Scale bar indicates 0.1 µm. B, Immunoelectron microscopic localization of Desi in the labellar sensillum of *Desi RNAi* fly using anti-Desi IgG. Scale bar indicates 0.1 µm.

Fig. 2. Immunoelectron microscopic analysis of Desi distribution in the larval epidermis. Broad (A) and narrow (B) scopes of immunoelectron microscopic localization of Desi in the epidermis of control *UAS-dsDesi* larva using anti-Desi IgG. Scale bar indicates 0.1 µm. Arrow heads indicate clusters of Desi immunopositive signals. C, Immunoelectron microscopic localization of Desi in the epidermis of control *UAS-dsDesi* larva using non-immunized IgG. Scale bar indicates 0.1 µm. D, Immunoelectron microscopic localization of Desi in the epidermis of *Desi RNAi* larva using anti-Desi IgG. Scale bar indicates 0.1 µm. EC: endocuticle, AZ: assembly zone, EpC: epidermal cell.

Fig. 3. Immunoelectron microscopic analysis of Desi distribution in the labellum epidermis. Broad and narrow scopes of immunoelectron microscopic localization of Desi in the longitudinal section of the labellum. Scale bar indicates 0.1 µm. EC: endocuticle, AZ: assembly zone, EpC: epidermal cell. Note that Desi immunopositive signals form small clusters in the assembly zone on the labellum epidermis as indicated by arrows.

Fig. 4. Immunoelectron microscopic analysis of Desi distribution in the labellum. A, Immunoelectron microscopic localization of Desi in the transverse section of the adult labellar sensillum at about half its length. Scale bar indicates 1 µm. B, Immunoelectron microscopic localization of Desi in the transverse section of the adult labellar sensillum beneath the endocuticle. Scale bar indicates 1 µm. Note that Desi immunopositive signals form small clusters in the sensillum lymph sinus as indicated by *.

Fig. 5. RT-PCR analysis of *Desi* expression levels. Typical RT-PCR result of *Desi*

expression levels in labella of control, *Desi* overexpression and RNAi flies (upper), and the quantitative RT-PCR results (lower). Data are given as means±SD for four separate measurements using 10 adults each. ** denotes $P<0.01$ relative to values under control *Desi*-Gal4 (Tukey's HSD).

Fig. 6. Scanning electron micrographs of labella of control and *Desi* RNAi flies. Scanning electron micrographs of labella of control (A, *Desi-Gal4*;*UAS-GFP*) and *Desi* RNAi flies (B, *Desi-Gal4*;*UAS-GFP* /*UAS-dsDesi*). Scale bars indicate 20 μm.

Fig. 7. Effect of *Desi* RNAi on gustatory neurons in the labellum. A, Distribution of sensory neurons (magenta) recognized by mouse anti-Futsch/22C10 monoclonal antibody in control (upper, *UAS-dsDesi*) and *Desi* RNAi (lower, *Desi-Gal4*; *UAS-dsDesi*) flies. Note that there is no significant morphological difference in neurons and the projection patterns between control and *Desi* RNAi lines. Scale bars indicate 20 μm. B, Expression of several neuron specific genes, including gustatory receptor genes, in the labella of control and *Desi* RNAi flies. Note that there is no difference in the gene expression levels between control and *Desi* RNAi lines except the *Desi* expression level.

.

Fig. 8. Feeding activities of *Desi* transgenic flies under 100% and 0% RH. A, Feeding activities of *Desi* overexpression flies under 100% RH. B, Feeding activities of *Desi* knockdown (RNAi) flies under 100% RH. C, Feeding activities of *Desi* overexpression flies under 0% RH. D, Feeding activities of *Desi* knockdown (RNAi) flies under 0% RH. 5mM sucrose solution was used as a diet. Data are given as means±SD for five separate measurements using 50 male adults each. ** denotes $P<0.01$ relative to values under control *Desi*-Gal4 (Tukey's HSD).

Fig. 9. Effect of *Desi* RNAi on survival rates of test *Drosophila* lines. Data are given means for three separate measurements using 50 animals each. Significant differences between control lines (*Desi-Gal4*(I) and *UAS-dsDesi*(III)) and *Desi* RNAi line were determined using generalized linear models.

Fig. 10. Effect of *TNT* overexpression in *Desi*-expressing cells on PER reaction of

adults. Proboscis extension reflex (PER) reaction was measured in *Drosophila* flies overexpressing *TNT* in *Desi*-expressing cells. Data are given as means±SD for five separate measurements using 35 adults each. Note that there is no difference between control and *TNT* overexpression lines.

Fig. 11. Effect of *Desi* RNAi on PER reaction of adults. Proboscis extension reflex (PER) reaction was measured in *Drosophila* flies repressed *Desi* expression in water (*ppk28*), sweet (*Gr5a*), and bitter (*Gr66a*) gustatory neurons. Data are given as means±SD for five separate measurements using 40 adults each. Note that there is no difference between control and *Desi* RNAi lines.

Supplementary Fig. 1

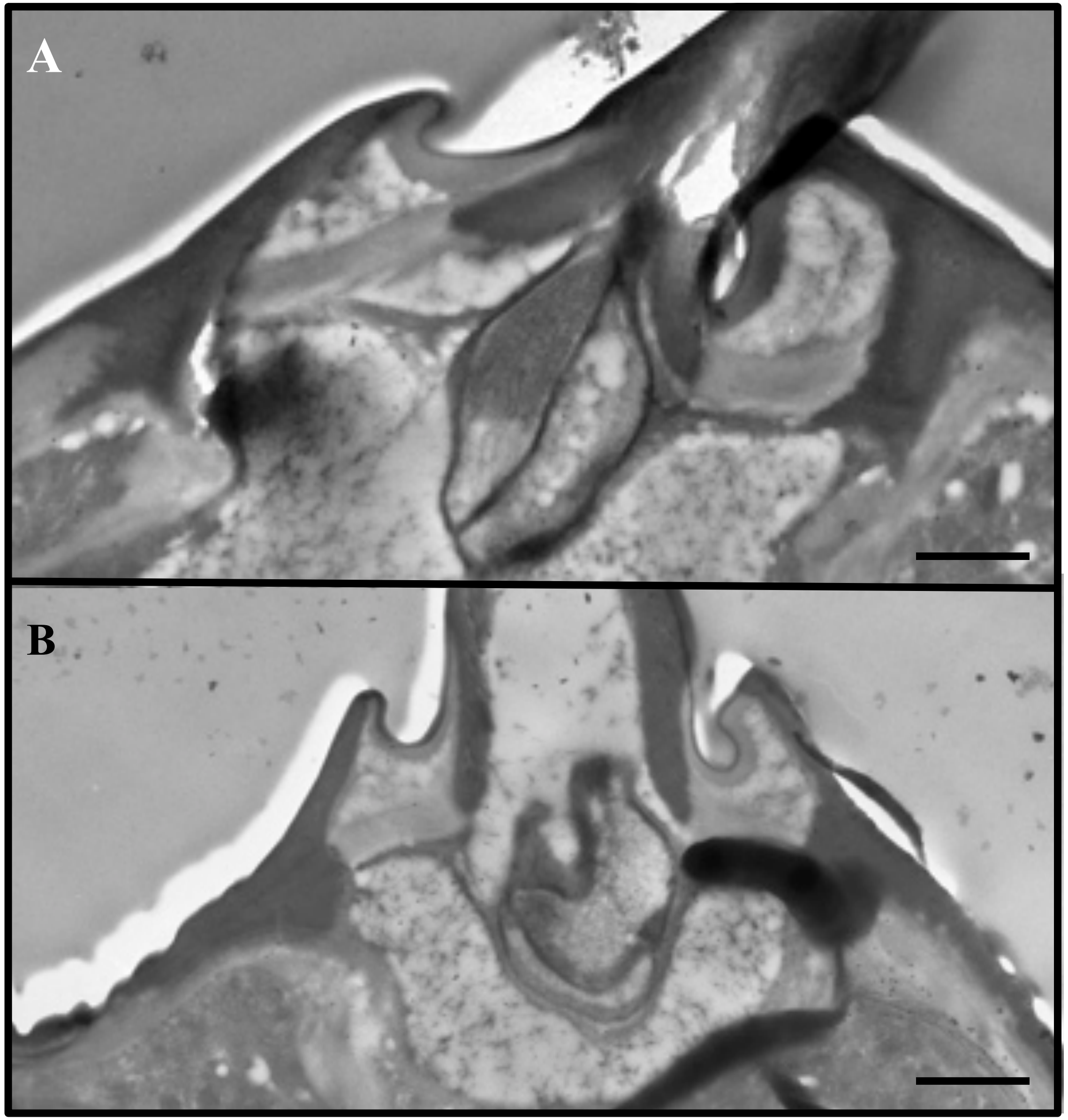

Supplementary Fig. 2

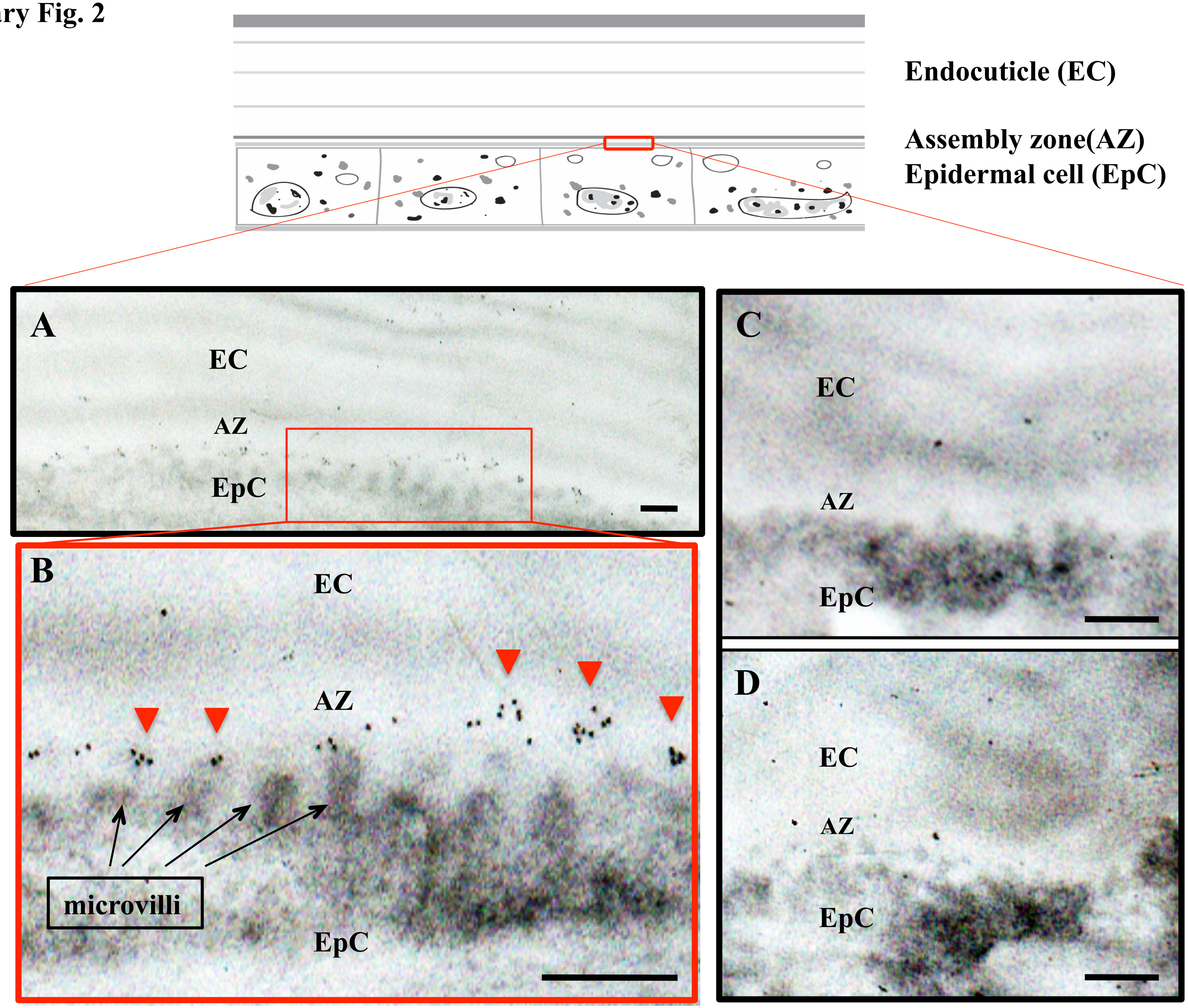

Supplementary Fig. 3

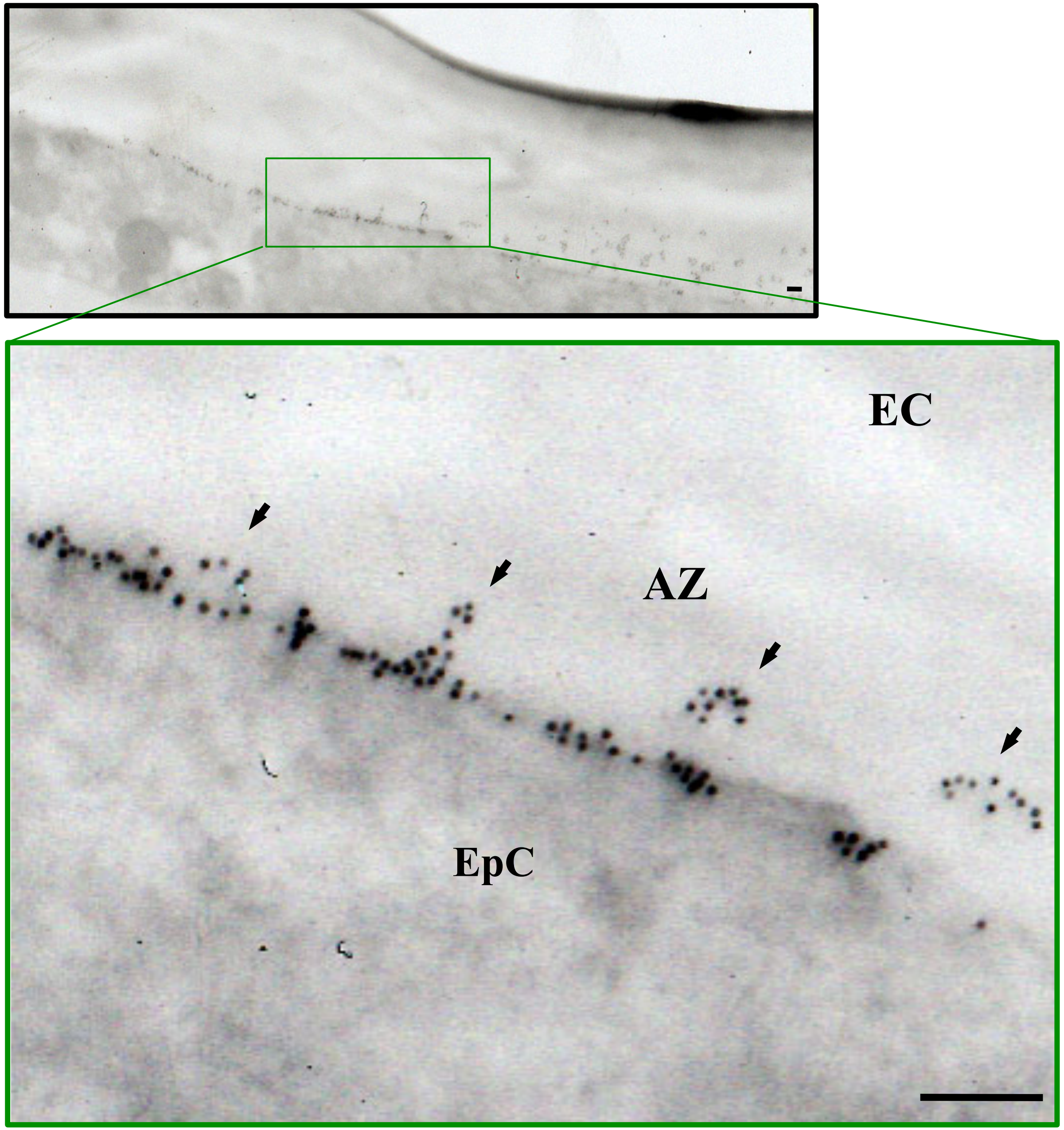

Supplementary Fig. 4

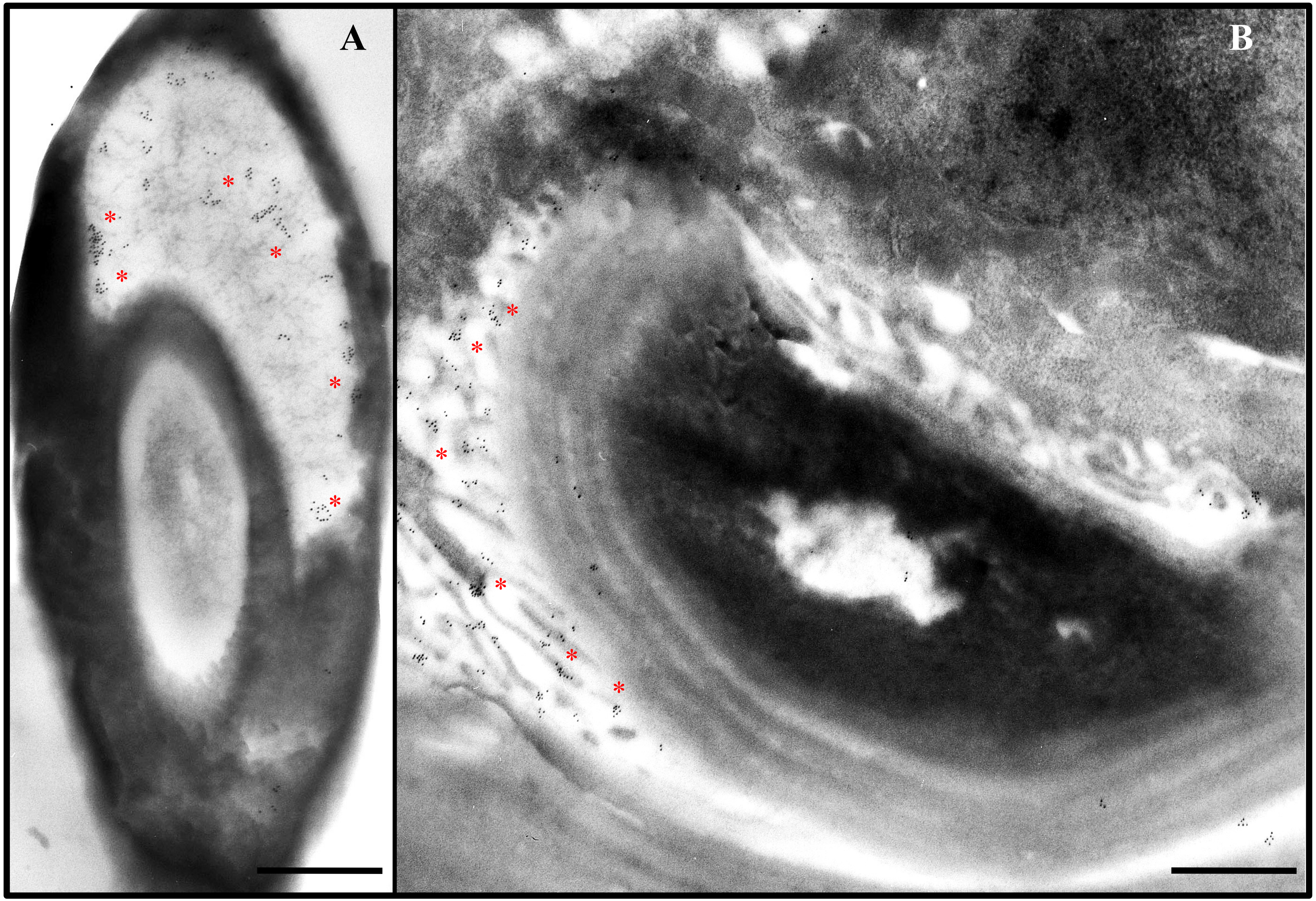

Supplementary Fig. 5

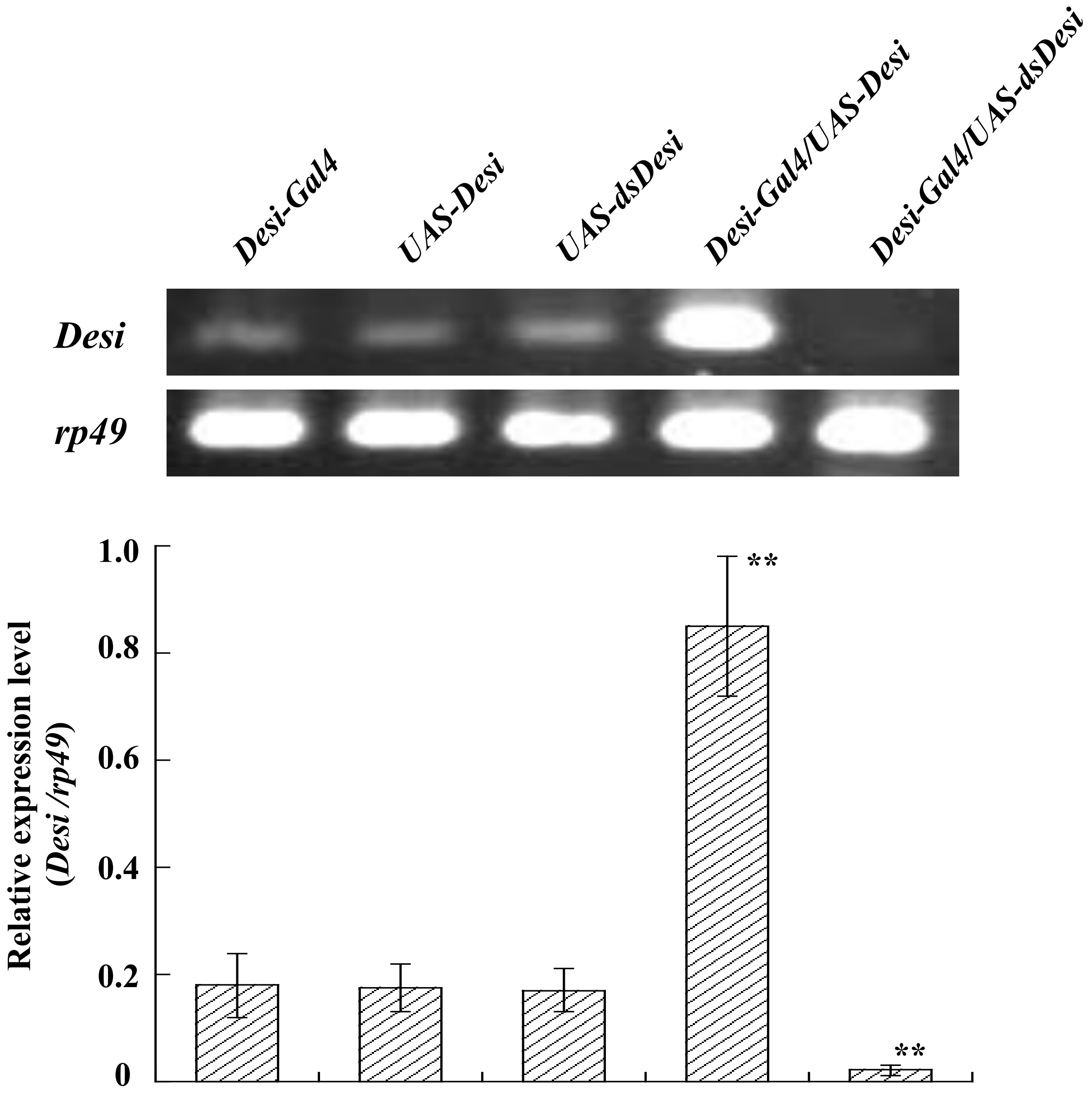

Supplementary Fig. 6

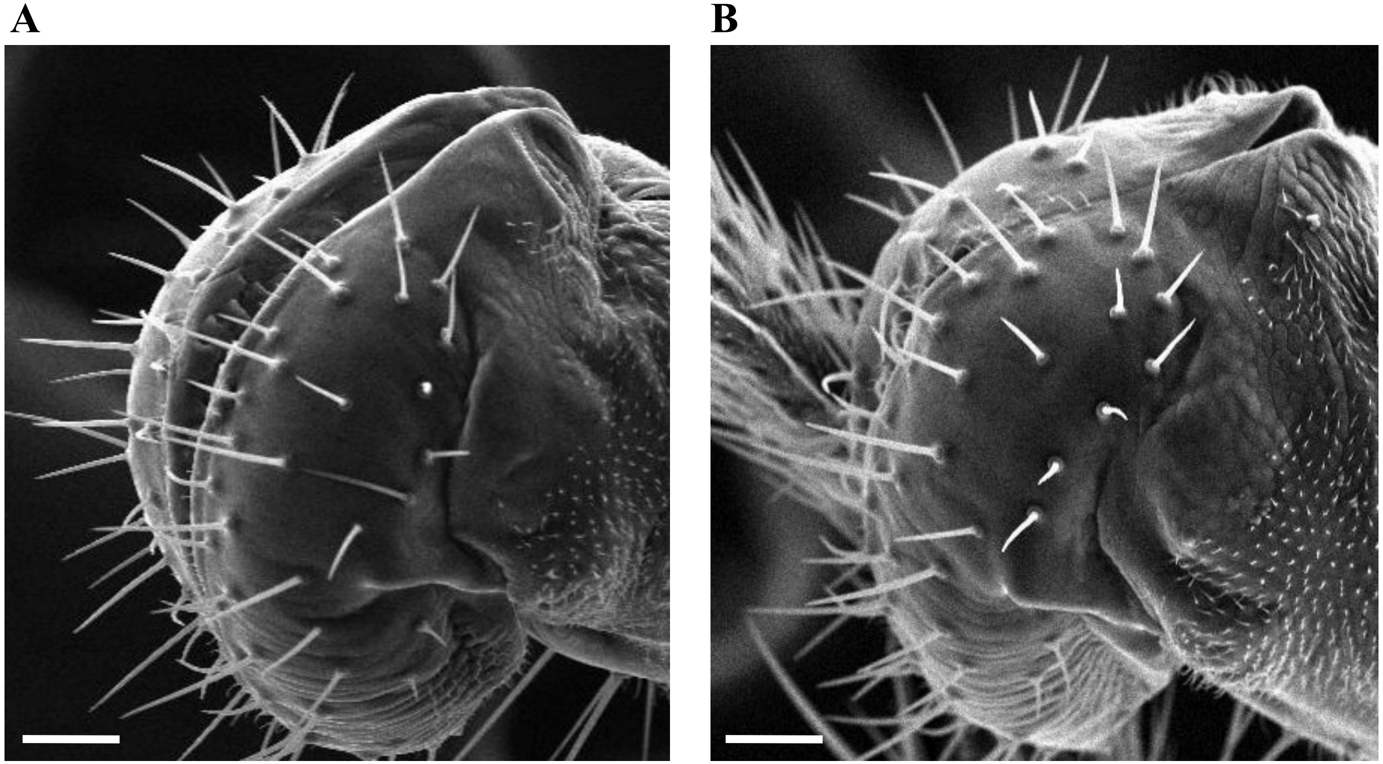

Supplementary Fig. 7

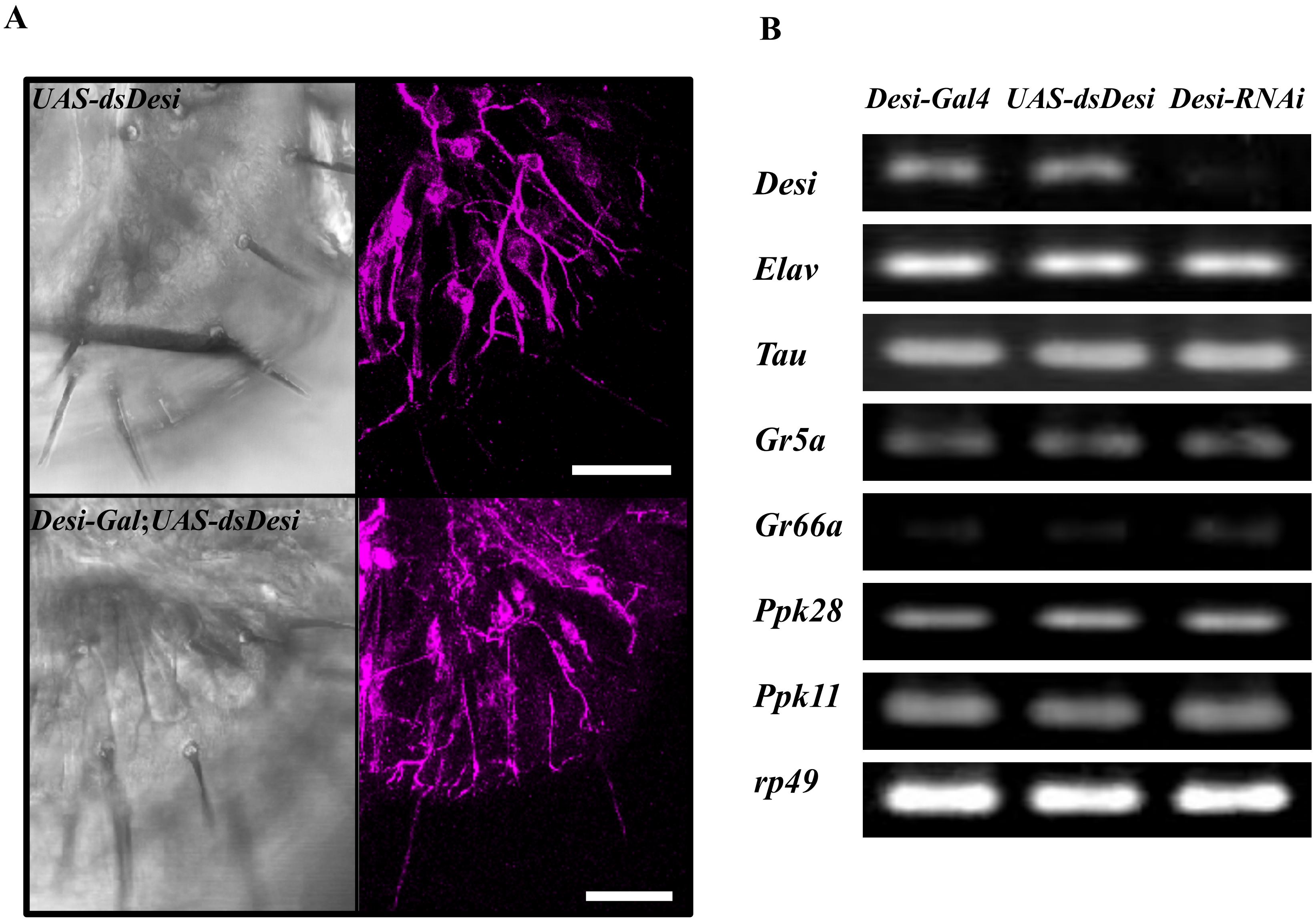

Supplementary Fig. 8

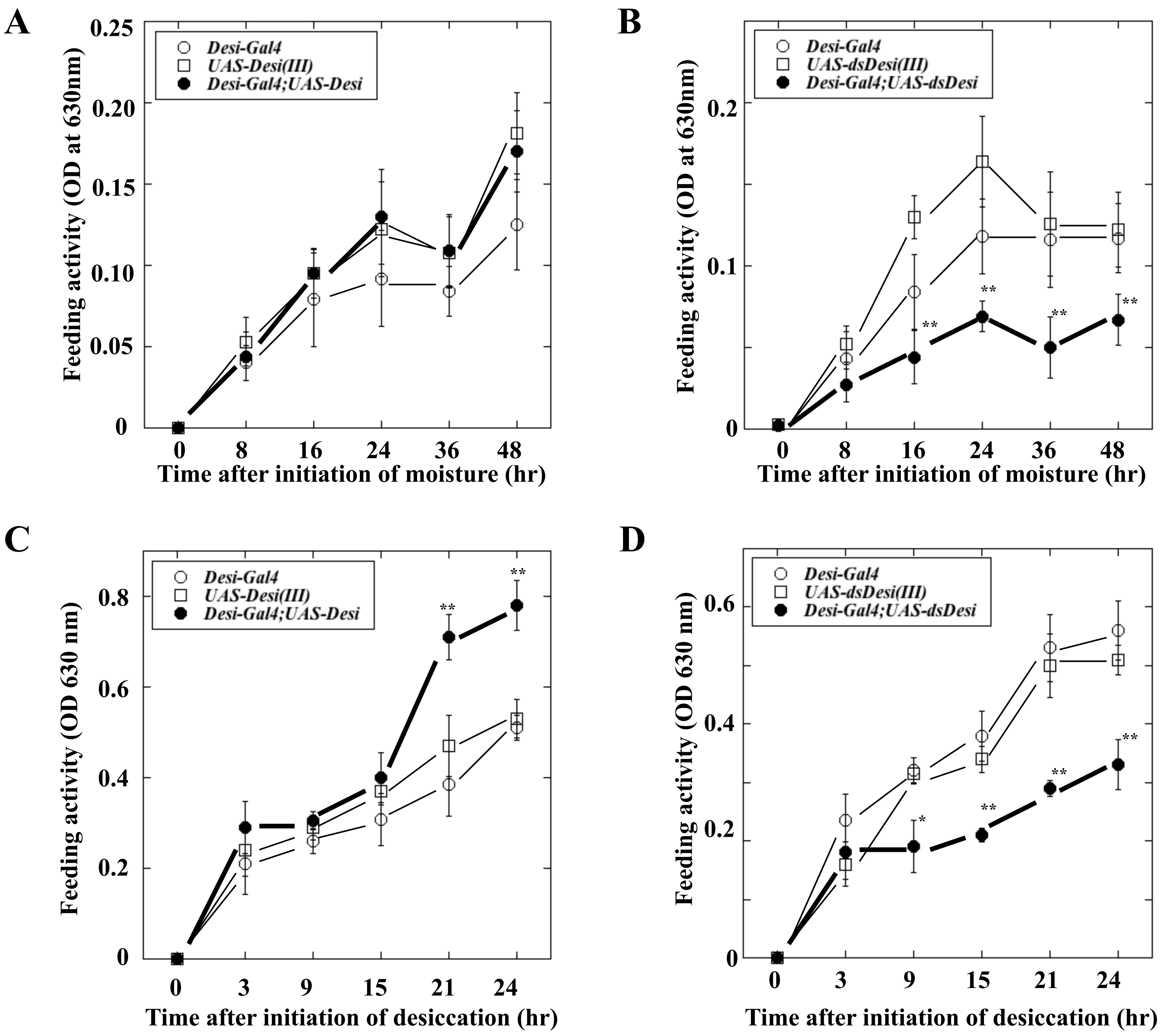

Supplementary Fig. 9

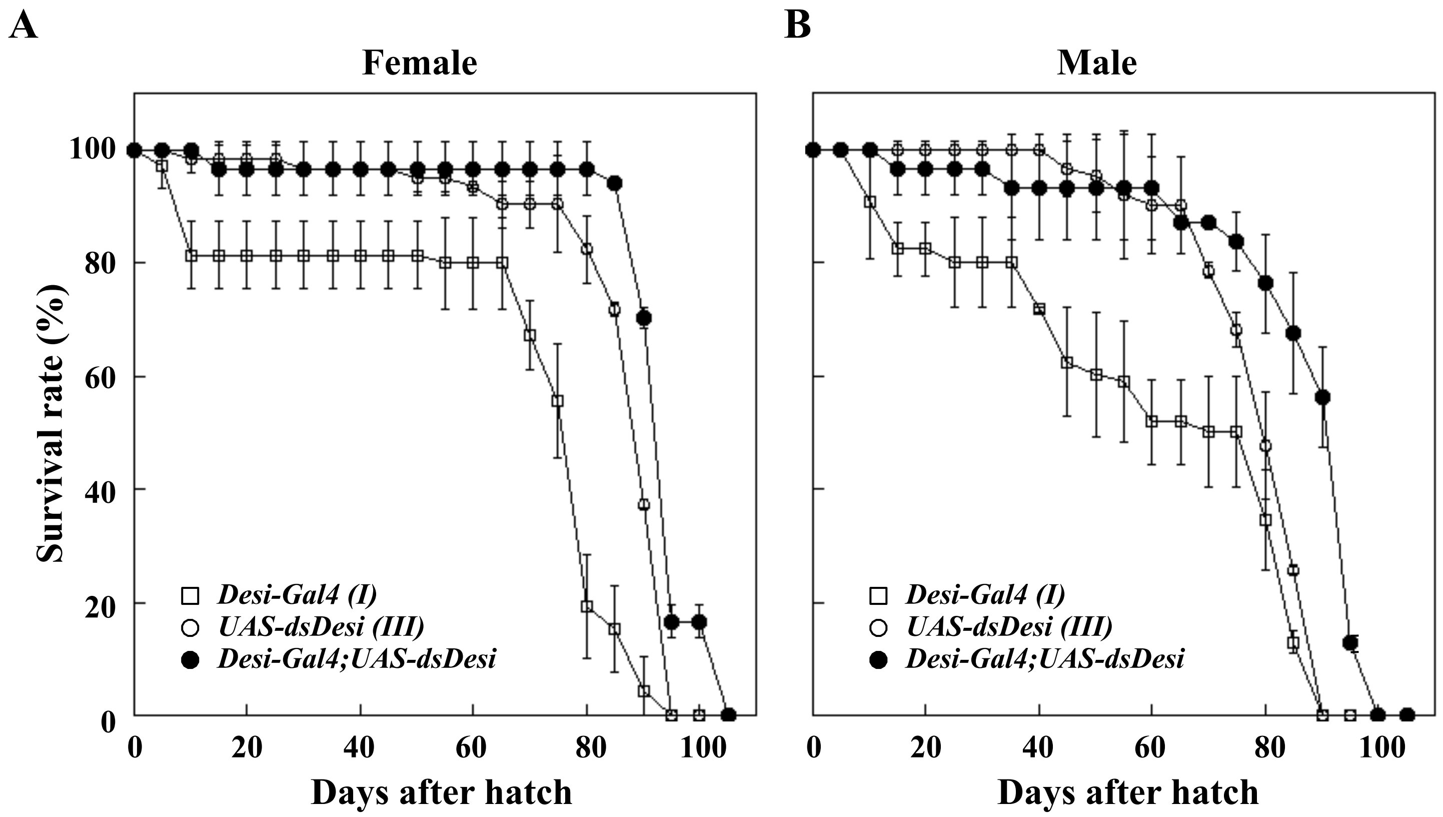

Supplementary Fig. 10

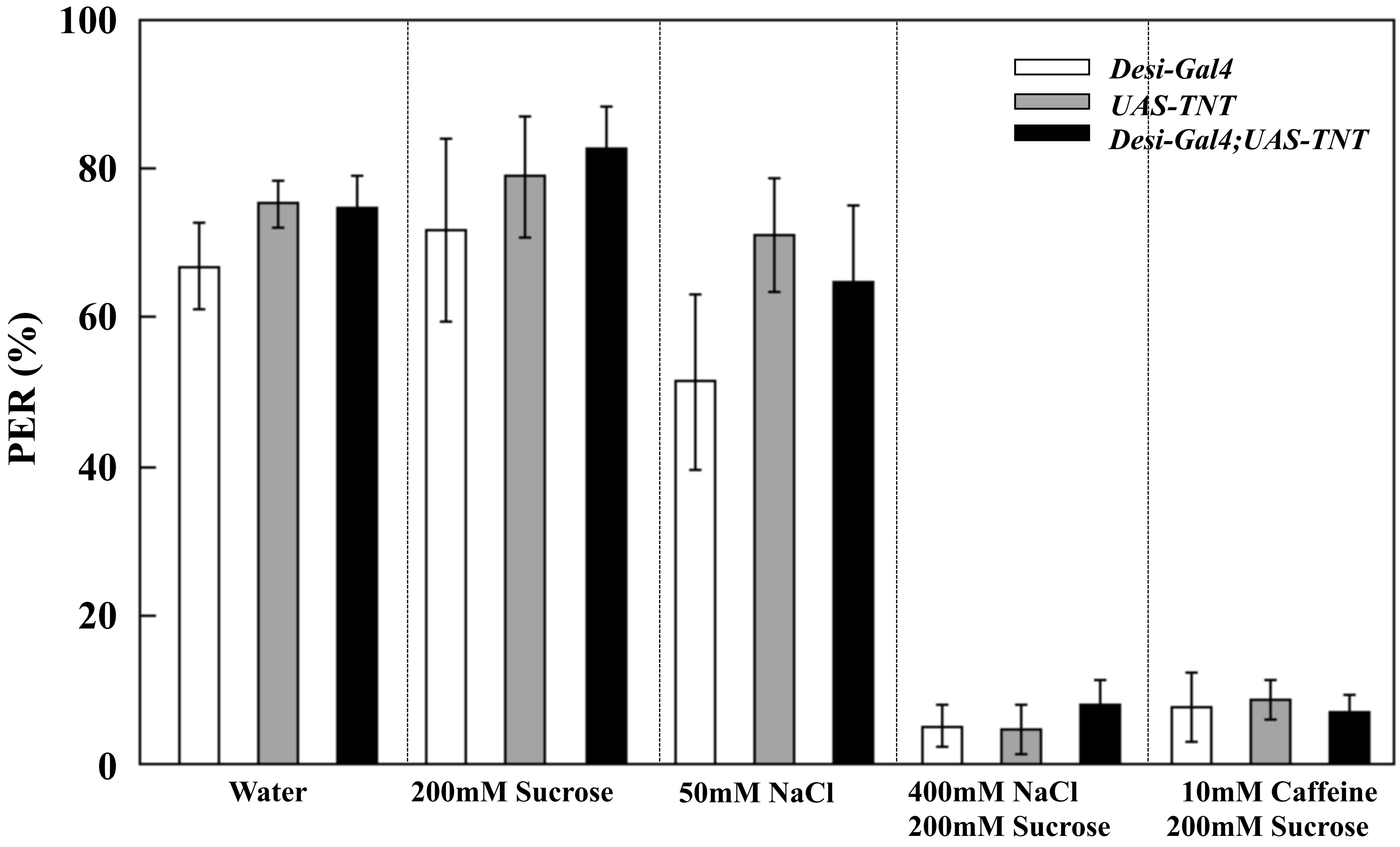

Supplementary Fig. 11

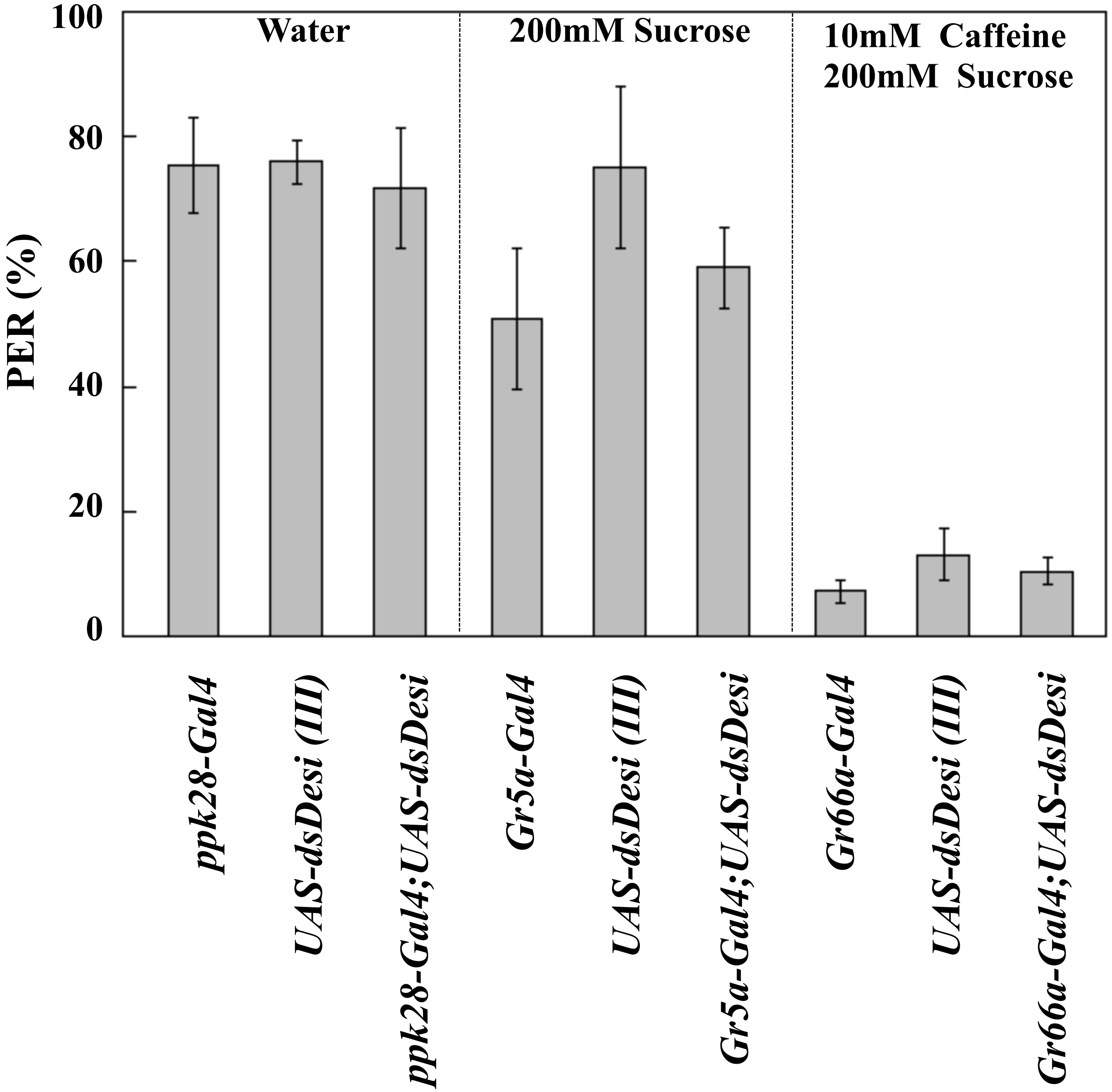

Supplement: Supplementary Information [file srep17195-s1.pdf]
